# Rapid Antibiotic Susceptibility Determination for *Yersinia pestis* Using Flow Cytometry Spectral Intensity Ratio (SIR) Fluorescence Analysis

**DOI:** 10.1007/s10895-018-2279-3

**Published:** 2018-08-16

**Authors:** Eran Zahavy, Shahar Rotem, David Gur, Ronit Aloni-Grinstein, Moshe Aftalion, Raphael Ber

**Affiliations:** Department of Biochemistry and Molecular Genetics, Israel Institute for Biological Research, Ness Ziona, Israel

**Keywords:** Bacterial fluorescence, Flow cytometry, Antibiotic susceptabilty test, *Yersinia pestis*

## Abstract

Rapid antimicrobial susceptibility tests (ASTs) are essential tool for proper treatment of patients infected by *Yersinia pestis* (*Y. pestis*), the causative agent of plague, or for post-exposure prophylaxis of a population exposed to a naturally acquired or deliberately prepared resistant variant. The standard AST of *Y. pestis* is based on bacterial growth and requires 24–48 h of incubation in addition to the time required for prior isolation of a bacterial culture from the clinical or environmental sample, which may take an additional 24–48 h. In this study, we present a new and rapid AST method based on a fluorescence determination of the minimum inhibitory concentration (MIC). Our method includes the incubation of bacteria with an antibiotic, followed by staining of the bacteria with oxonol dye (SynaptoGreen C4/FM1–43), which enables the rapid detection of an antibiotic’s effect on bacterial viability. We show that stained, non-viable bacteria exhibit a spectral redshift and an increase in fluorescence intensity compared to intact control bacteria. Based on these criteria, we developed a rapid flow cytometer measurement procedure and a unique spectral intensity ratio (SIR) analysis that enables determination of antibiotic susceptibility for *Y. pestis* within 6 h instead of the 24 to 48 h required for the standard AST. This new rapid determination of antibiotic susceptibility could be crucial for reducing mortality and preventing the spread of disease.

The development of rapid antimicrobial susceptibility tests (ASTs) is a major concern given the current state of global increase in antibiotics resistance. Antibiotic resistant pathogens, either naturally occurring due to extensive medical and agricultural use of antibiotics, or intentionally developed as bio-terror agents, are an increasing threat to public health. Hence, proper medical care requires the development of new rapid methods to identify bacteria and determine their susceptibility to the antibiotic treatment of choice, as empirical therapy based only on infective agent identification may not always be effective. The need for rapid ASTs is most urgent for in vitro slow growing bacteria with the capability to develop rapid, acute disease. *Yersinia pestis*, (*Y. pestis*), the causative agent of plague, is classified by the CDC as a Tier 1 select agent (http//www.bt.cdc.gov/agent/agentlist-catagory.asp) and is a representative of such infectious agents. Inhalation of this agent results in rapidly progressing pneumonic plague that can be transmitted from person to person. High mortality rates are observed if treatment does not start within 18–24 h after the onset of symptoms [1–3]. Unfortunately, the current standard AST method for *Y. pestis* (CLSI) [4] requires 24–48 h, not including the time required for bacterial culture isolation.

A variety of methods for ASTs have been developed [5]; some are based on improved agar-diffusion tests such as the “Etest” [6–8]; others are based on novel automated monitoring technologies such as the determination of growth by plate-readers [5, 9], digital time-lapse microscopy [10], microscopic observation of colony formation [11], using immune-labeled bacteria and growth curve by FCM [12], statistical analysis to un labeled bacteria post cleaning steps [13], laser light scattering methods [14, 15] or pre-enrichment methods [16]. Others have introduced methods to monitor biological changes such as an output of the antibiotic treatment. The latter includes measurement of the immediate bacterial transcriptome response to antibiotic agents [17], Raman spectroscopy for emergent biomarkers [18] and various fluorescent markers [19–25]. A rapid fluorescence/flow-cytometry AST method was reported by Nuding et al. [23], who monitored bacterial viability by measuring membrane potential using the oxonol dye DiBAC_4_ as a fluorophore indicator. Interestingly, although each antibiotic agent leads to a different inactivation path, using a membrane potential probe enables prediction of bacterial viability at the end of the course. However, this method requires prolonged incubation of dye with the bacteria and extensive washing steps. Moreover, the exact prediction of the bacterial state using fluorescent labels and flow cytometry analysis of a variety of conditions that cause cell death, including antibiotics, requires a more complex combination of fluorophores; thus, a single dye is not sufficient for all inactivation methods [26, 27]. Furthermore, since different antibiotic agents trigger different antibacterial mechanisms such as cell wall damage, protein synthesis inhibition or DNA destruction, leading to vast bacterial response mechanisms, the use of a single dye detector is not trivial.

In this work, we present a new fluorescence-based prediction method for bacterial viability determination based on flow cytometry using the lipophilic fluorescent dye SynaptoGreen C4 (FM1–43) as a new tool for rapid AST. This dye exhibits spectral sensitivity to either a lipophilic or hydrophilic chemical environment through a solvent relaxation effect [28] (Fig. 1a) and self-quenching [29, 30]. A correlation between the bacterial viability state and the spectral behavior of the dye was shown for detection of viable bacteria in water [31, 32]. For live bacteria, the dye stained the lipophilic membrane and exhibited weak fluorescence, while in bacteria killed by either UV, bleach or heat, the fluorophore changed its position from the intact membrane to the cytoplasm of the inactivated bacteria, resulting in a “red” spectral shift zone and stronger fluorescence, as presented schematically in Fig. 1a. This “red” spectral shift for inactivated bacteria compared to live bacteria is mainly attributed to the shift of the fluorophore from the membrane structure in live bacteria to the cytoplasm upon bacterial inactivation. This shift changes the fluorophore environment from the hydrophobic surroundings of the membrane to the hydrophilic surroundings of the cytoplasm, leading to the solvent relaxation effect as described in Fig. 1b [28]. The changes in the intensity and the position of the fluorescence spectrum on the wavelength axis allows for a calculation of a spectral intensity ratio (SIR), as shown in Eq. 1. By calculating the ratio between the SIR of treated bacterial samples to the SIR of a live control sample, where the SIR of inactive bacteria is greater than the live bacteria, one can determine the viability of the bacteria in the unknown sample and discriminate between live and dead bacteria.

**Fig. 1:**
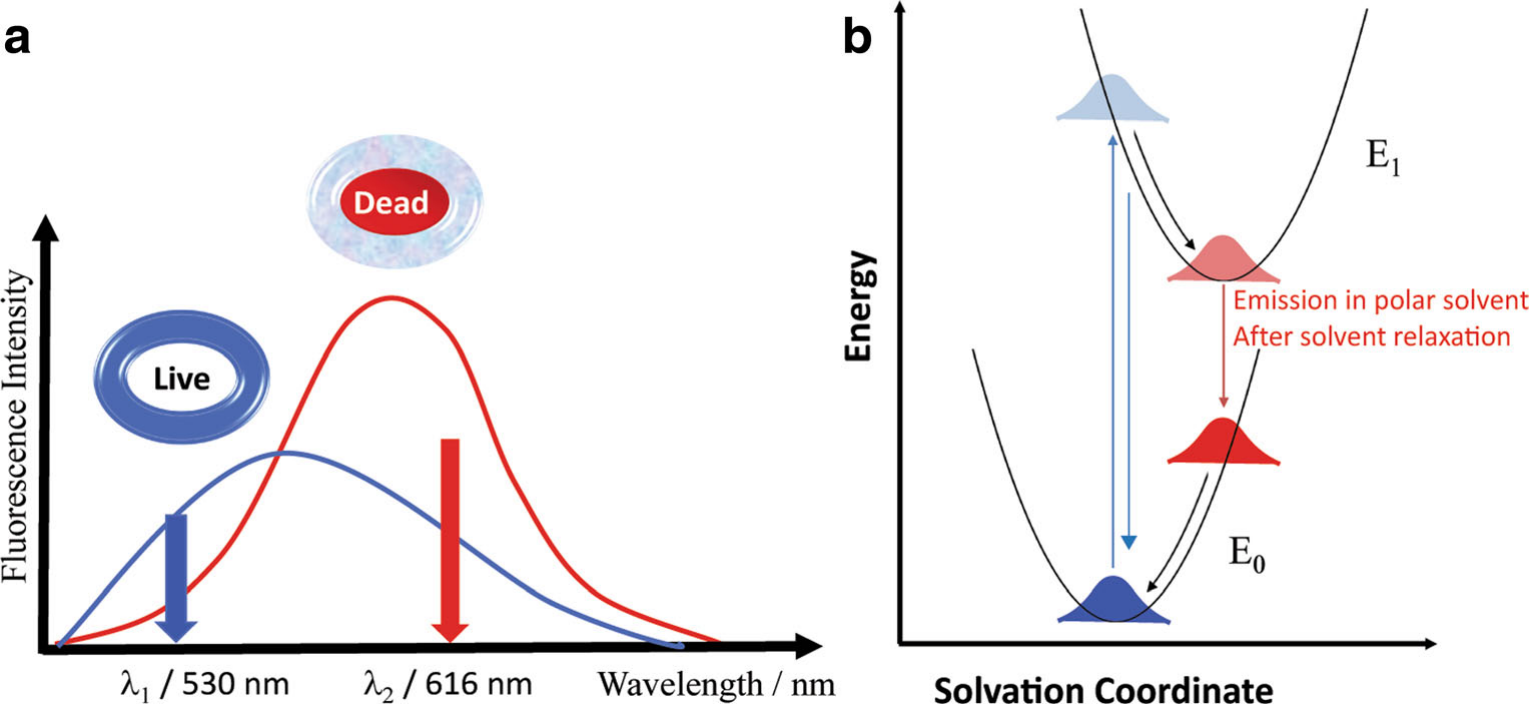
Schematic fluorescence spectra of SynaptoGreen (FM1–43) stained live (blue curve) vs. killed (red curve) bacteria, illustrating spectral shift and intensity change (SIR) behavior. Live bacteria in blue, inactivated in red. λ_1_ and λ_2_ used for SIR calculation

Based on these data, we hypothesized that employing a fluorescence/flow-cytometry and SIR analysis on a single-bacterium level would allow the estimation of the physiological response to antibacterial agents, permitting rapid detection of bacterial susceptibility to the antibiotic, with a significantly reduced assay time for *Y. pestis*.

1 $$ \boldsymbol{SIR}=\frac{{\boldsymbol{Intensity}}_{\left({\uplambda}_{\mathbf{2}}=\mathbf{616}\ \boldsymbol{nm}\right)}}{{\boldsymbol{Intensity}}_{\left({\uplambda}_{\mathbf{1}}=\mathbf{530}\ \boldsymbol{nm}\right)}} $$

## Material and Methods

### Bacterial Strains and Growth Conditions

Bacterial strains used in this work are the *Y. pestis* vaccine strain EV76 [33], the plasmid-cured non-virulent *Y. pestis* Kimberley53∆pPCP1∆pCD1 (Kim53Δ10Δ70) strain [34] and its reduced ciprofloxacin sensitive and non-sensitive sub species Kim53Δ10Δ70–42 and Kim53Δ10Δ70–66-6 respectively [2, 3, 17]. Doxycycline reduced sensitivity and resistant sub species are Kim53Δ10Δ70–36 and Kim53Δ10Δ70–36-4 respectively. All strains were plated on BHI-A plates (brain heart infusion agar, BD Difco, Sparks, MD U.S.A. #241830) and incubated at 28 °C. Colony-forming unit (CFU) counts were determined in duplicates by plating 100 μl of serial ten-fold dilutions in sterile phosphate-buffered saline (PBS, Biological Industries, Beit Haemek, Israel) on BHI-A plates. Drop-plating was performed by plating 10 μl of serial ten-fold dilutions in triplicates on BHI-A plates.

### Antibiotic Susceptibility Tests

ASTs were performed using the standard microdilution method [35] using ciprofloxacin solution (Ciproxin 200, Bayer), ampicillin (Sigma A0166), gentamicin (Sigma G1264) and doxycycline (Sigma D9891). For susceptibility tests, stock solutions were serially diluted two-fold in cation-adjusted Müeller-Hinton broth (CAMHB, BBL 212322) and placed in 96-well flat bottom microtiter plates (TPP 92696). 50 μl of freshly prepared bacterial suspensions in CAMHB were inoculated in triplicates with 5*10^5^-1*10^6^ CFU/ml in a final volume of 0.1 ml containing antibiotic concentrations in the range of 0.03–16 × MIC. Cultures were incubated for 24 h at 28 °C in a plate reader (Sunrise or Infinite F-200 pro, TECAN), and the optical density at 630 nm (OD630) was read at 1-h intervals. The MIC values were defined after 24 h as the lowest antibiotic concentration that reduced growth to less than 10% of the OD at 630 nm of the growth control (without antibiotics). Lack of growth in MIC wells was verified visually by naked eye inspection. Each assay was performed in three independent experiments. For the time-lapse susceptibility analysis by FACS, similar AST conditions were used in a 24-well flat bottom microplate (Costar #3524) in a final assay volume of 0.5 ml.

### Bacteria Inactivation

Inactivated *Y. pestis* EV76 and Kim53Δ10Δ70 were suspended in PBS to approximately 1*10^8^ CFU/ml. Ethanol inactivation was performed by exposure of 3*10^7^ CFU aliquots to 70% ethanol for 15 min. Heat inactivation was achieved by exposing 5*10^5^ CFU aliquots in a volume of 50 μl to 70 °C for 40 minutes. Dry-inactivation was achieved by an exposure of 5*10^5^ CFU aliquots in a volume of 50 μl PBS to aseptic ventilation at 37 °C for 24 h. After liquid evaporation, the original volume was restored with sterile dH_2_O. Inactivation by all preparation methods was verified by plating on BHI-A plates and incubation for 3 days at 28 °C. All inactivated samples were stored at 4 °C until use.

### Dyes

Membrane dyes SynaptoGreen C4 (Sigma, S6814) or Fm1–43 (Invitrogen, T3163) were prepared in PBS and used for bacterial labeling at 5 μM. Fluorescence spectra of live and dead bacteria were measured in a TECAN Infinite M-200 pro plate reader.

***Flow-Cytometry analysis*** of bacteria [36] was performed on a FACSAria III from Becton Dickinson (San Jose, CA) using a 488-nm laser for excitation and band path emission filters 530 (±15) nm, marked as 530/30, and 616 (±11.5) nm, marked as 616/23, from BD. For analysis, both Flow-Jo and FCS Express software were used for calculation and graphic display, respectively. The SIR was calculated from the ratio between the mean signals measured at 616 nm and at 530 nm. The SDL parameter was calculated as the ratio between the number of events representing dead bacteria to live bacteria multiplied by SIR, Eq. 2.

2 $$ \mathrm{SDL}=\mathrm{SIR}\times \left(\mathrm{Dead}/\mathrm{Live}\right) $$

MIC calculation by FCM: The SIR multiplied by the Dead to Live ratio (SDL) values were calculated and plotted vs. antibiotic concentration. The average of at least 4 samples plus 5 times the standard deviations of SDL values of live bacteria with no antibiotic addition is calculated as the threshold between live and dead bacteria. The first antibiotic concentration exhibiting SDL level higher than the threshold is determined as the MIC.

## Results

**Fig. 2:**
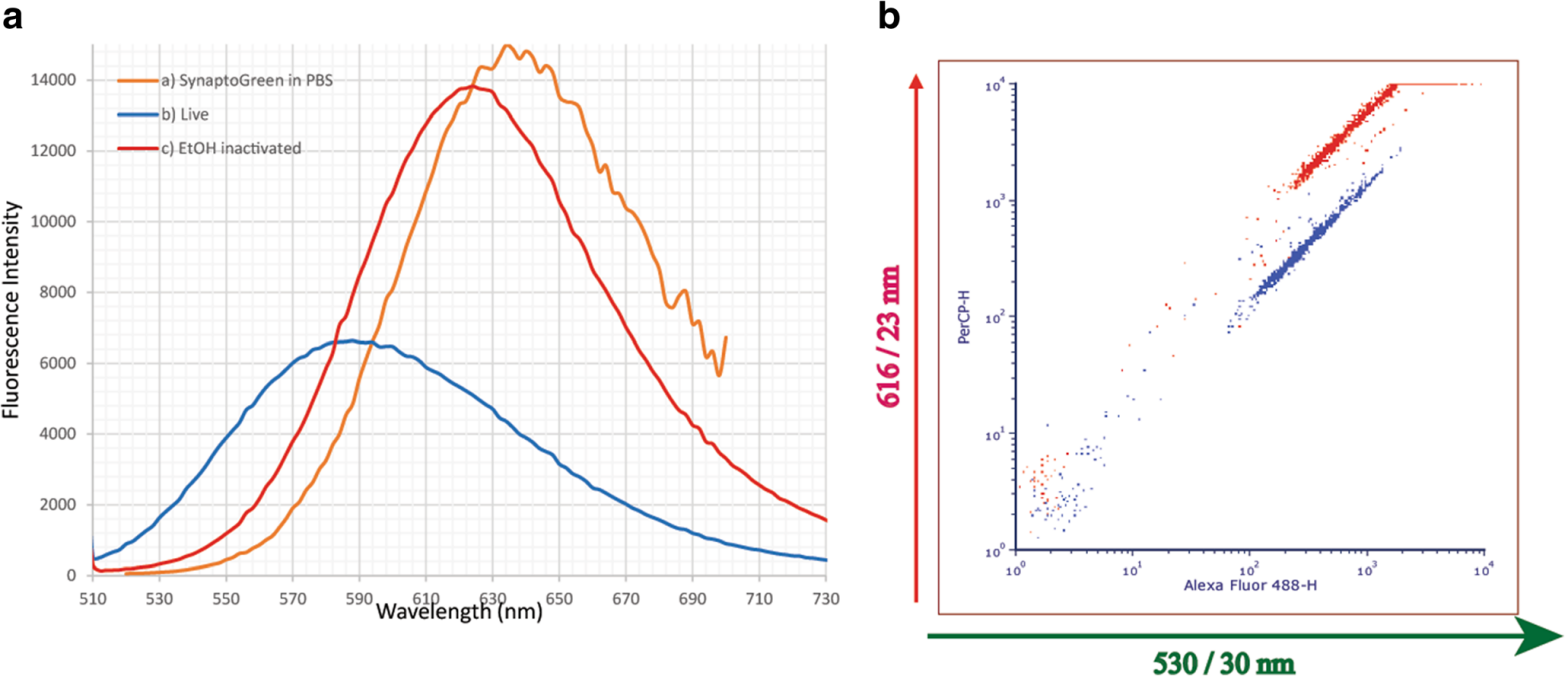
**a** Fluorescence spectral analysis of SynaptoGreen in buffer (orange curve), in live *Y. pestis* EV76 (blue curve) and ethanol-inactivated EV76 (red curve). Bacteria conc. 1*10^8^ CFU/ml. **b** Flow cytometry fluorescence dot plot, 616/23 nm vs. 530/30 nm, of *Y. pestis* EV76 mixture of live (blue) and ethanol-inactivated (red) after SynaptoGreen staining, 5 μM. Bacteria conc. 1*10^6^ cfu/ml

To assess the SIR method as a tool for determination of antibiotic sensitivity of *Y. pestis*, we first verified the basic SIR concept on live versus ethanol-inactivated *Y. pestis* bacteria (Fig. 2). The spectra consist of overlay curves of SynaptoGreen in PBS (orange curve), stained live *Y. pestis* (EV76) bacteria (blue curve) and stained ethanol-inactivated bacteria (red curve). Upon staining of live bacteria with the membrane dye SynaptoGreen, there is a shift in the fluorophore emission spectrum to a shorter wavelength (“blueshift”) compared to its free form in PBS. In the fluorescence curve for ethanol-killed *Y. pestis* bacteria (Fig. 2a, red curve) the fluorescence maximum is shifted to 624 nm compared to the live bacteria peak at 590 nm (blue curve), or to 638 nm where the dye is in PBS (orange curve). The overall fluorescence of inactivated bacteria compared to live bacteria, is more intense and “redshifted”. The “redshift” can be attributed to the hydrophilic nature of the cytoplasm, resulting in a “relaxation effect” of the dye’s electronic states; the increased intensity can be attributed to more dye entering the cell cytoplasm and averting the self-quenching process between the dye’s electronic states within the membrane. The result allows a clear distinction between live and dead bacteria, which exhibits stronger emission and is “redshifted”. Two wavelengths, λ_1_ and λ_2_, are used to calculate the SIR values for each bacterium in Eq. 1. From the fluorescence spectral shift, one can notice two distinct wavelengths that reflect the spectral shifts at 616 nm and 530 nm (Fig. 2a). Hence, the SIR value is calculated by the fluorescence intensity ratio of 616 nm over 530 nm. The SIR values of the live and dead bacteria were calculated to be 3.5 and 40, respectively.

Measuring such effect using standard fluorimeter which measures the whole sample is not easy to apply, since it involves a concentration dependence on the effect. Meaning that variation in concentration between samples will alter results and increase noise. Hence it is preferable to use single cell (bacteria) fluorescence measurement methods such as cell scan, scanning microscopy or flow cytometry (filter or spectral).

We have shown that the same phenomenon can be used in a flow cytometry (FCM) system that enables a single-bacterium analysis and high-throughput screening. The possibility of measuring effects on a single-bacterium level is crucial because it diminishes the dependence on a fixed concentration, as in bulk fluorescence measurements, and it increases sensitivity.

Figure 2b shows an FCM dot plot of the designated fluorescence filters, corresponding to two emission wavelengths chosen for the SIR effect: λ_1_ at 530/30 nm (530 ± 15 nm) and λ_2_ at 616/23 nm (616 ± 11.5 nm). SIR is calculated using the flow cytometry statistics tool, as the mean fluorescence at the 616 nm filter over the mean fluorescence at the 530 nm filter, following Eq. 1: SIR_FCM_ = mean FL(616) /mean FL(530). The sample contains a mixture of live and inactivated bacteria treated with ethanol and stained with SynaptoGreen. Using control samples, where live and inactivated bacteria were separated, we could define the live bacteria gate (blue) and inactivated bacteria gate (red). The SIR values of the live and dead bacteria were calculated to be 2.5 and 15, respectively. From the FCM and fluorescence spectra, we determined that SIR _dead_ > SIR _live_, where the difference in values may be attributed to differences in measurement methods. These data ensured the consistency of the SIR effect for *Y. pestis* and prompted us to examine its utilization as an early marker for MIC determination and rapid AST.

**Fig. 3:**
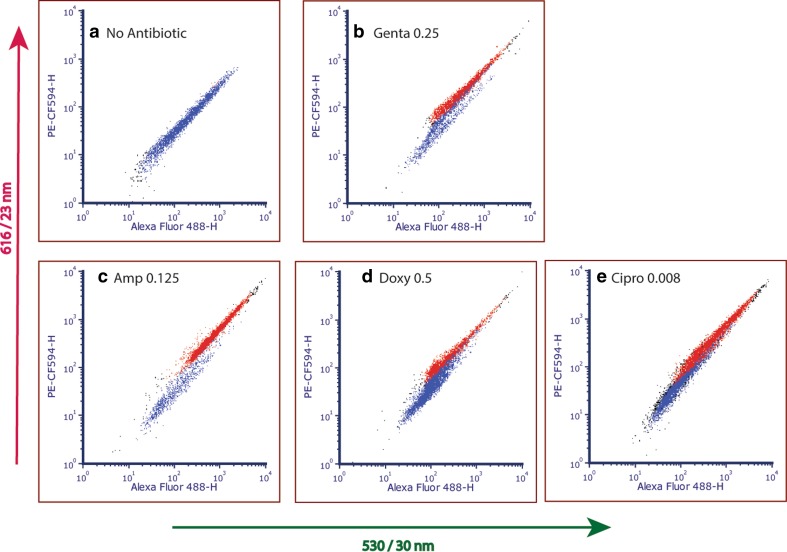
Fluorescence dot plots, 616/23 nm vs. 530/30 nm, of *Y. pestis* EV76 with SynaptoGreen staining for SIR analysis. **a** Growth control with no antibiotic, **b** Gentamicin 0.25 μg/ml, **c** Ampicillin 0.125 μg/ml, **d** Doxycycline 0.5 μg/ml and **e** Ciprofloxacin 0.008 μg/ml

To utilize our method for MIC determination on *Y. pestis*, we next tested the validity of the SIR method following antibiotic treatment. To that end, *Y. pestis* EV76 bacteria were exposed to several antibiotic agents in a series of two-fold dilution concentrations, and the change in SIR was measured at different incubation time points and compared to the standard AST of 24 h incubation period. The antibiotic effect on bacterial staining with respect to the SIR change and ratio between designated live and dead bacteria is presented in Fig. 3, showing the fluorescence FCM dot plot of *Y. pestis* bacteria grown for 10 h under standard growth control medium (CAMHB with no antibiotic treatment, Fig. 3a). As this dot plot represents a live bacterial population, it was designated by the FCM gate as blue dots, while the dead bacterial population was designated in a different gate as red dots, as shown in Fig. 2b. The SIR value for the live bacteria (Fig. 3a) was calculated from the whole dot plot to be 0.3(±0.1). Figures 3b-e depict fluorescence dot plots of *Y. pestis* EV76 incubated in the presence of a minimum inhibitory concentration of the examined antibiotics after 10 h of exposure. These dot plots show an increase in the number of “red” events, which is attributed to the dead bacteria gate, and an overall increase in SIR values, as summarized in Table 1. Overall, compared to the control sample with no antibiotic (Fig. 3a) or to sub-MIC concentrations (data not shown), it is clear that under sufficient antibiotic concentrations (i.e., at the MIC value and higher) there is an increase in both the SIR and the percentage of dead bacteria in the culture population. From the SIR _treatment_/SIR _growth control_ ratio, the increased SIR under antibiotic treatment is notably less imminent than under ethanol treatment or other physical and chemical inactivation methods such as heat or dryness (data not shown). This is likely due to more complex time-concentration dependent physiological death/growth-arrest mechanisms imposed by the antibiotic agents [37]. However, the dot plots of treated bacteria shown in Figs. 3b-e, clearly show a dead bacteria (red dots) population.

**Table 1:** Results and analysis of SIR/SDL flow cytometry for *Y. pestis* EV76 under various antibiotic treatments, as shown in Fig. 3

	MIC (μg/ml)	Dead (%)	Live (%)	D/L ratio	SIR*	SIR _treat_/SIR _live_	SDL*	SDL_treat_/SDL_live_
No antibiotic	–	0.5	99	0.005	0.3	1	0.0015	1
Gentamicin	0.25	62	28	2.2	0.53	1.76	1.2	778
Ampicillin	0.125	83	12	6.9	0.59	1.96	4	2714
Doxycycline	0.5	33	74	0.45	0.46	1.53	0.21	138
Ciprofloxacin	0.008	48	50	1	0.6	2	0.6	400
Ethanol		98	1	98	15	6	1470	980,000

*Experiments repeated 3 to 4 times, yielding standard error of 10 to 15%

To increase the criteria for the determination of damaged/inactive bacteria over noise, we multiplied the SIR values with the ratio of the “dead bacteria/live bacteria” (D/L) as evaluated by the FCM gate statistics. It is important to note that these are two different parameters; where SIR indicates the change in the dye and the gate statistic indicates the change in the bacteria population. This resulted in a new representative value of SDL, Eq. 2. The values obtained using SDL calculations resulted in higher signal-to-noise values, as seen in Table 1. Therefore, we obtained a more sensitive indication of the bacterial viability state, and hence a more sensitive determination of MIC.

**Fig. 4:**
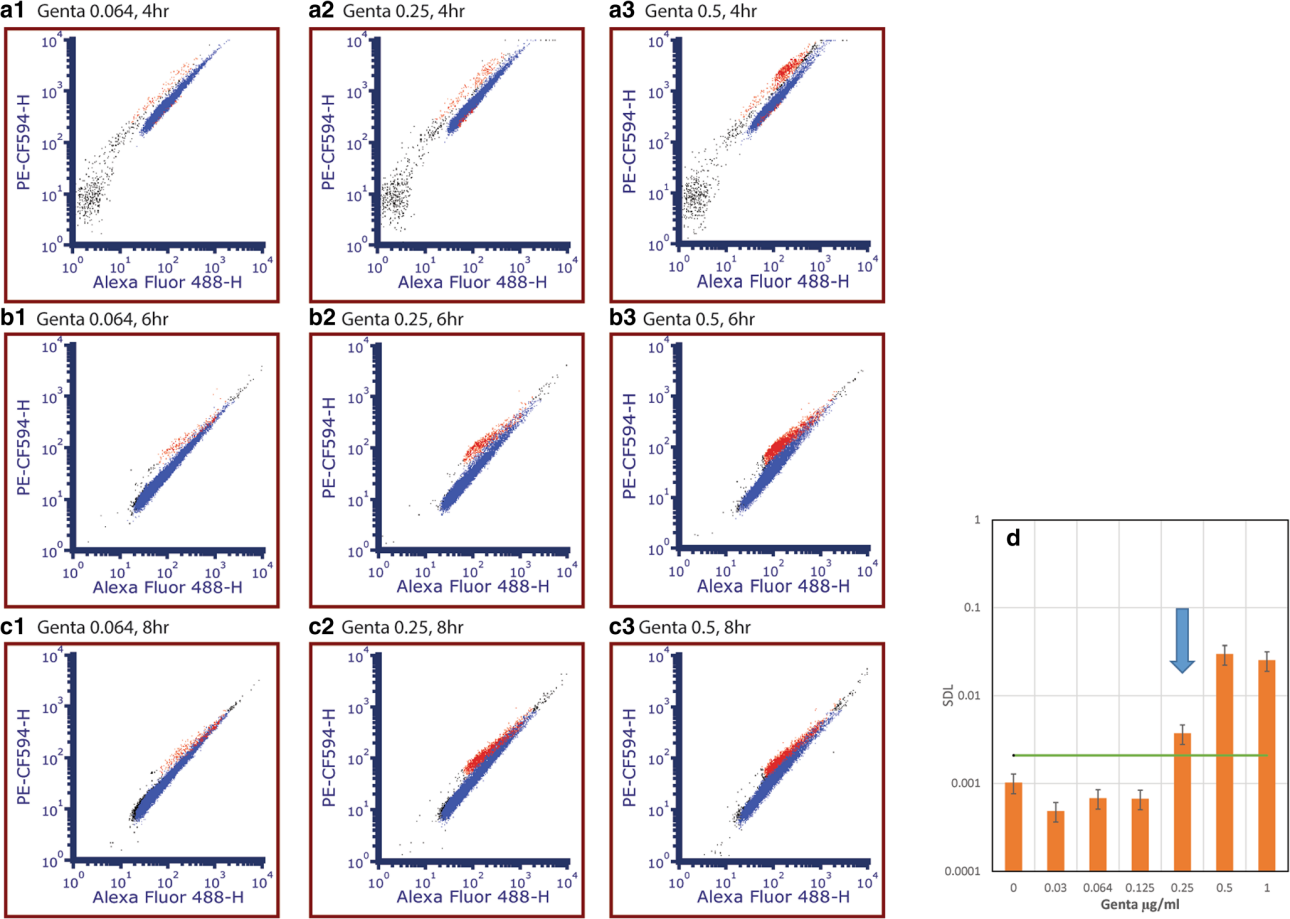
Flow cytometry fluorescence dot plots for SIR analysis of *Y. pestis* (EV76) under Gentamicin treatment for **a** 4 h, **b** 6 h, **c** 8 h under sub MIC, 0.064 μg/ml (1) MIC, 0.25 μg/ml (2) and supra MIC, 0.5 μg/ml, and (3) exposure conditions. **d** SDL calculated from the 6 h time lap, green line is the calculated threshold from 6 samples of live bacteria with no antibiotic in the same condition. At 0.25 μg/ml of gentamicin the SDL represent the MIC value

To determine the minimal incubation time required for bacteria to be affected by an antibiotic prior to SIR reading and D/L calculation, microdilution tests of *Y. pestis* EV76 were conducted in time increments of 2, 4, 6, 8, 10 and 24 h of antibiotic exposure. Figure 4 represents an example of the flow-cytometry SIR and SDL analysis of the stained SynaptoGreen bacteria under gentamicin exposure at various exposure times (4, 6, and 8 h) at sub-MIC (0.064 μg/ml), MIC (0.25 μg/ml) and supra-MIC concentrations (0.5 μg/ml). After 4 h of gentamicin exposure, at a sub-MIC concentration of 0.064 μg/ml (Fig. 4 A1), there is no indication of inactivated bacteria (red dots), where both SIR and the percentage of red dots is <0.5%, similar to growth control. Indication of a rise in the inactivated bacteria fraction appears in 0.5 μg/ml, which is only one two-fold dilution above the standard MIC (0.25 μg/ml). After 6 h of gentamicin exposure, the same phenomenon appeared in the culture exposed to 0.25 μg/ml gentamicin, which is the MIC measured under standard microdilution conditions. The collective dot plots from such an experiment, including the calculated values of the mean fluorescence intensity of both fluorescence channel (for SIR calculation) and the dead/live population percentage is processed to achieve SDL values per time and per antibiotic concentration. Such analysis is presented in the bar graph in Fig. 4d. To calculate where the threshold line that above it non-viable bacteria population will be defined, a statistic calculation of the average noise plus 5 times STDEV is derived from measurements of 4 samples with no antibiotic exposure. One can observe that at the antibiotic (gentamycin) concentration of 0.25 μg/ml we can define dead bacteria population, hence a MIC value of 0.25 is defined.

**Fig. 5:**
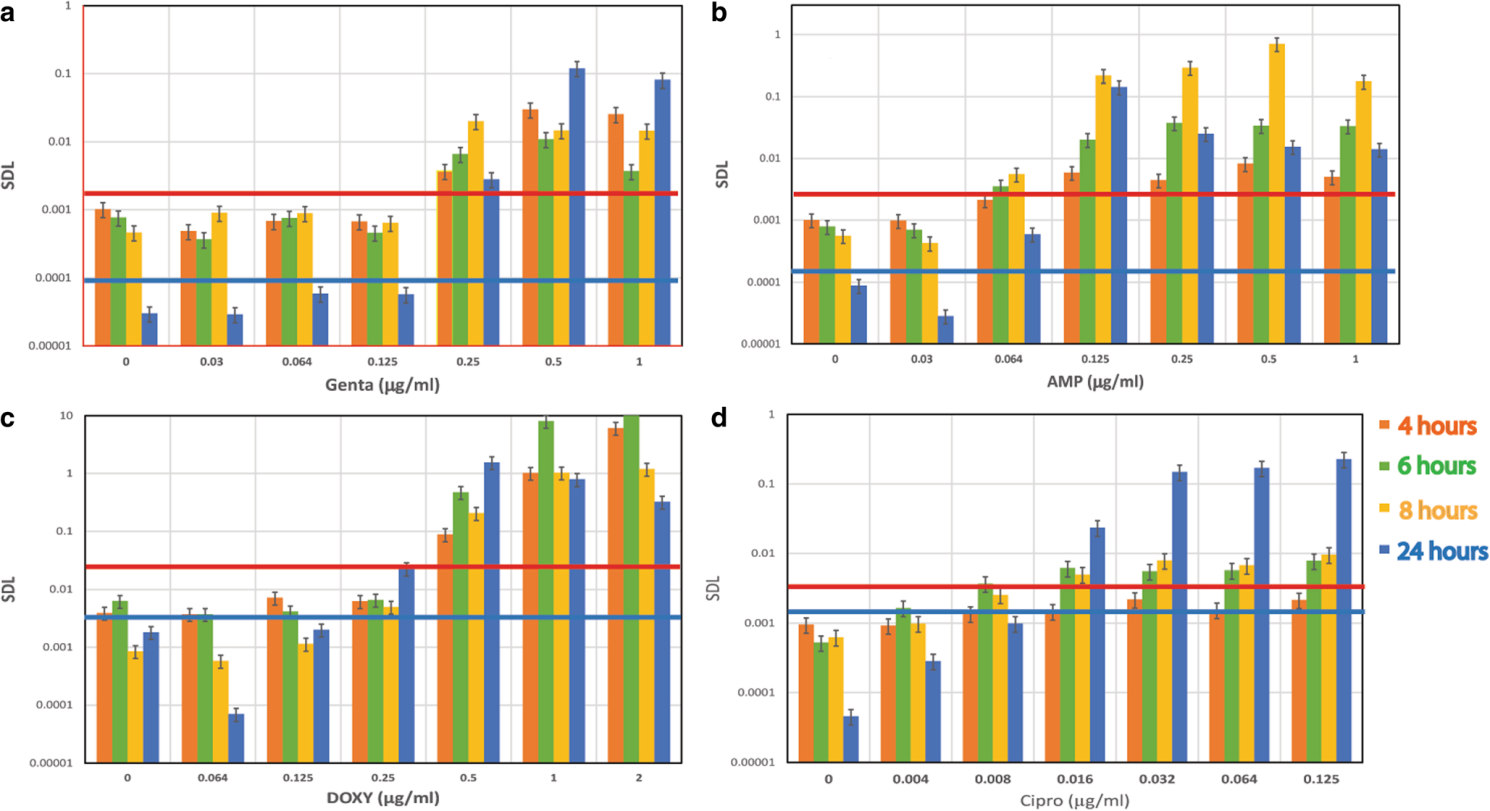
*Y. pestis* (EV76) SDL values per time-laps (4, 6, 8, 24 h.) and antibiotic concentration as measured by SDL method for: **a** Gentamicin, **b** Ampicillin, **c** Doxycycline, and **d** Ciprofloxacin. MIC determination is calculated by the first antibiotic concentration where SDL exceeds the threshold level, as described in M&M. In here threshold line in red is for time laps of 4, 6 and 8 h, blue is the line for 24 h. Threshold line for 24 h is significantly reduced due to high rise of bacterial counting for non-antibiotic samples as control

The quantitative data for such experiments was similarly collected for four different antibiotic agents: gentamicin, ampicillin, doxycycline and ciprofloxacin. Their full two-fold dilution concentration panels were used for MIC evaluation and repeated at several time increments: 2, 4, 6, 8, 10 h, and the standard incubation period of 24 h. Fig. 5 shows the outcome of *Y. pestis* EV76 bacteria SDL values (on a logarithmic scale) versus four tested antibiotics: A. gentamicin, B. ampicillin, C. doxycycline and D. ciprofloxacin, at various concentrations over different exposure times (4, 6, 8 and 24 h). The SDL values as a function of gentamicin concentration at varying exposure times are shown in Fig. 5a. The MIC for each time interval is derived as the minimum antibiotic concentration where SDL is greater than the calculated threshold performed by the statistic of non-antibiotic samples as described above. Table 2 summarizes all evaluated MIC values at different time increments compared to a standard exposure period of 24 h. The data shows that at gentamicin concentrations of 0.5 μg/ml, after 4 h of incubation, the SDL value increased in a step-wise fashion to a value greater than the threshold, indicating a MIC value of 0.5 μg/ml. Similarly, we can determine MIC values of 0.25 μg/ml after 6, 8, 10 and 24 h (Table 2). All MIC values are comparable to standard MIC determinations within one dilution (well) separation using the CLSI method [35], in compliance with FDA instructions for evaluation of new susceptibility methods [38]. Although comparable gentamicin MICs are obtained by SDL and the standard microdilution methods, there is a marked difference in the time required to obtain the MIC using the SDL method; the SDL method took only 4 h compared to the standard method requiring 24 h. Moreover, although the MIC value deviates by one dilution, the correct category of sensitive bacteria is still achieved, as summarized in Table 3. An exposure to ampicillin (Fig. 5b) following analysis with the SDL method resulted in MIC values of 0.125 μg/ml, which is comparable (equal to single or double dilution) to the MIC (0.125 μg/ml) obtained using the standard method. This observation could also be obtained within 4 h of exposure instead of 24 h. In the doxycycline AST shown in Fig. 5c, the SDL method enabled correct MIC determination after 4 h of exposure, revealing the correct MIC of 0.5 μg/ml. Upon exposure to ciprofloxacin, we observed the correct MIC value of 0.008–0.016 μg/ml, within an early antibiotic exposure time of 4–6 h. All obtained MIC values in the experiments were compared to standard MIC measurements using the standard microdilution method, data not shown. In all assays we were able to derive the correct MIC, within one dilution, and were able to interpret the correct sensitivity category as shown in Table 3.

**Table 2:** MIC values for *Y. pestis* strains with different antibiotics and time exposure based on SDL method compared to standard microdilution

*Y. pestis* strains	Antibiotic	MIC Calculated from SDL at time laps of:					Standard MIC by 24 h AST
		4 h	6 h	8 h	10 h	24 h	
EV76^S^	Gentamycin	**0.5**	0.25				0.25
EV76^S^	Ampicillin	0.125	0.064–0.125				0.125
EV76^S^	Ciprofloxacin	ND	**0.008**–0.016				0.016
Kim53Δ10Δ70^S^		ND	[0.04]-**0.08**				0.016
Kim53Δ10Δ70–42, Cip^RS^		ND	**0.064**				0.125
Kim53Δ10Δ70–66-6, Cip^NS^		ND	16–32				16
EV76^S^	Doxycycline	0.5	0.5–0.25				0.5
Kim53Δ10Δ70–36, Dox^RS^		ND	**4**				2
Kim53Δ10Δ70–36-4, Dox^R^		ND	16–32				16

Superscripts: S, RS, NS and R, stand for sensitive, reduced sensitivity, non-sensitive and resistant accordingly

ND stands for Not Determined* **In bold**: MIC values one well (one two-fold dilution) or less apart from standard microdilution test

**In brackets**: MIC values two wells apart from microdilution test

**Table 3:** Category definition for the various *Y. pestis* strains as measured by SDL method compared to the CLSI category

*Y. pestis* strains	Antibiotic	Category interpretation by SDL:					24 h standard AST	Categories definition by CLSI*
		4 h	6 h	8 h	10 h	24 h		
EV76^S^	Gentamycin	S	S				S	S: MIC ≤4 I: MIC = 8 R: MIC ≥16
EV76^S^	Ampicillin	NA	NA				NA	NA
EV76^S^	Ciprofloxacin	ND	S				S	S: MIC ≤0.25 Not Sensitive (NS) at MIC >0.25
Kim53Δ10Δ70^S^		ND	S				S	
Kim53Δ10Δ70–42, Cip^RS^		ND	S				S	
Kim53Δ10Δ70–66-6, Cip^NS^		ND	NS				NS	
EV76^S^	Doxycycline	S	S				S	S: MIC ≤4 I: MIC = 8 R: MIC ≥16
Kim53Δ10Δ70–36, Dox^RS^		ND	S				S	
Kim53Δ10Δ70–36-4, Dox^R^		ND	R				R	

*S/I/NS/R stand for: Sensitive, Intermediate, Non-Sensitive and Resistant

*ND* stands for Not Determined, *NA* stands for Not Applicable

**Fig. 6:**
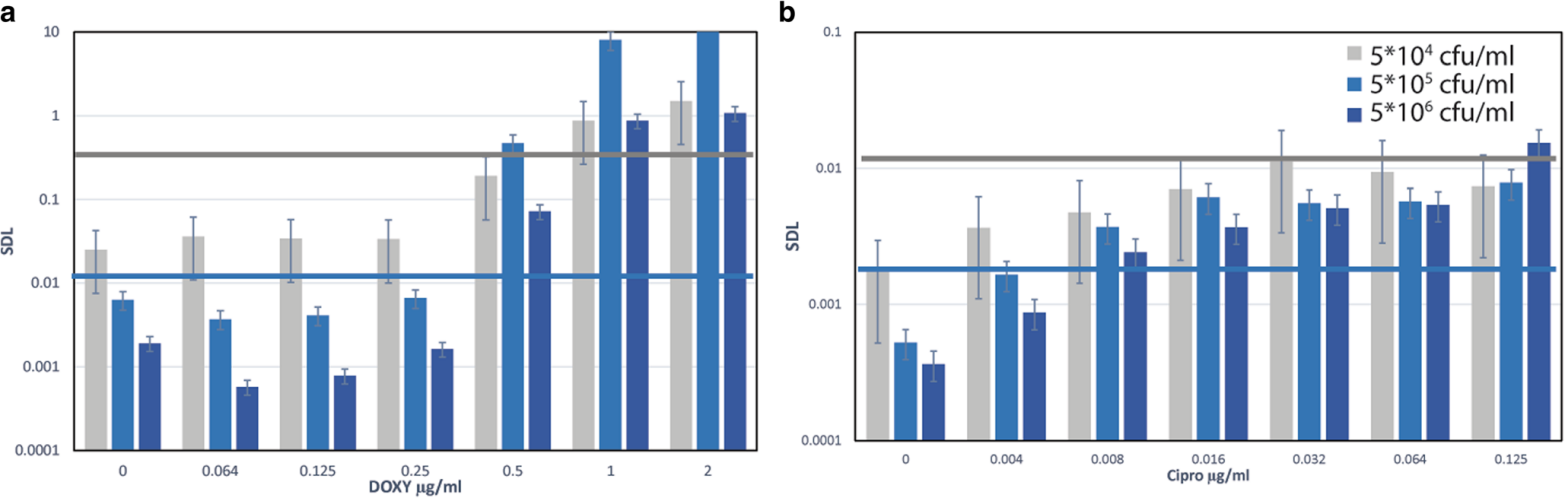
*Y. pestis* (EV76) SDL values after 6 h of incubation at several initial inoculum concentration of 5*10^4^ (gray), 5*10^5^ (light blue) and 5*10^6^ cfu/ml (darker blue). **a** Doxycycline and **b** Ciprofloxacin measurements. Using the threshold method, MIC is derived in the 5*10^5^ and 5*10^6^ cfu/ml initial concentration. Gray line is the threshold for the lower 5*10^4^ cfu/ml, blue line is the calculated threshold for the higher concentrations (5*10^5^ and 5*10^6^ cfu/ml) experiments.

All the experiments mentioned above were performed using the initial bacteria concentration as required by CLSI guidelines. To measure the initial bacteria concentration dependency of the assay we have used three different initial inoculum concentrations: 5*10^4^, 5*10^5^ and 4*10^6^ cfu/ml, where the 5*10^5^ cfu/ml is the recomended concentration for *Y. pestis* AST by the CLSI. As shown in Fig. 6 one can see that in both higher concentrations of 5*10^5^ and 5*10^6^ cfu/ml, we can observe the MIC at the same time lapses. However using a low initial concentration of 5*10^4^ cfu/ml, results in non-defined results, probably due to high flow cytometry reading noise.

**Fig. 7:**
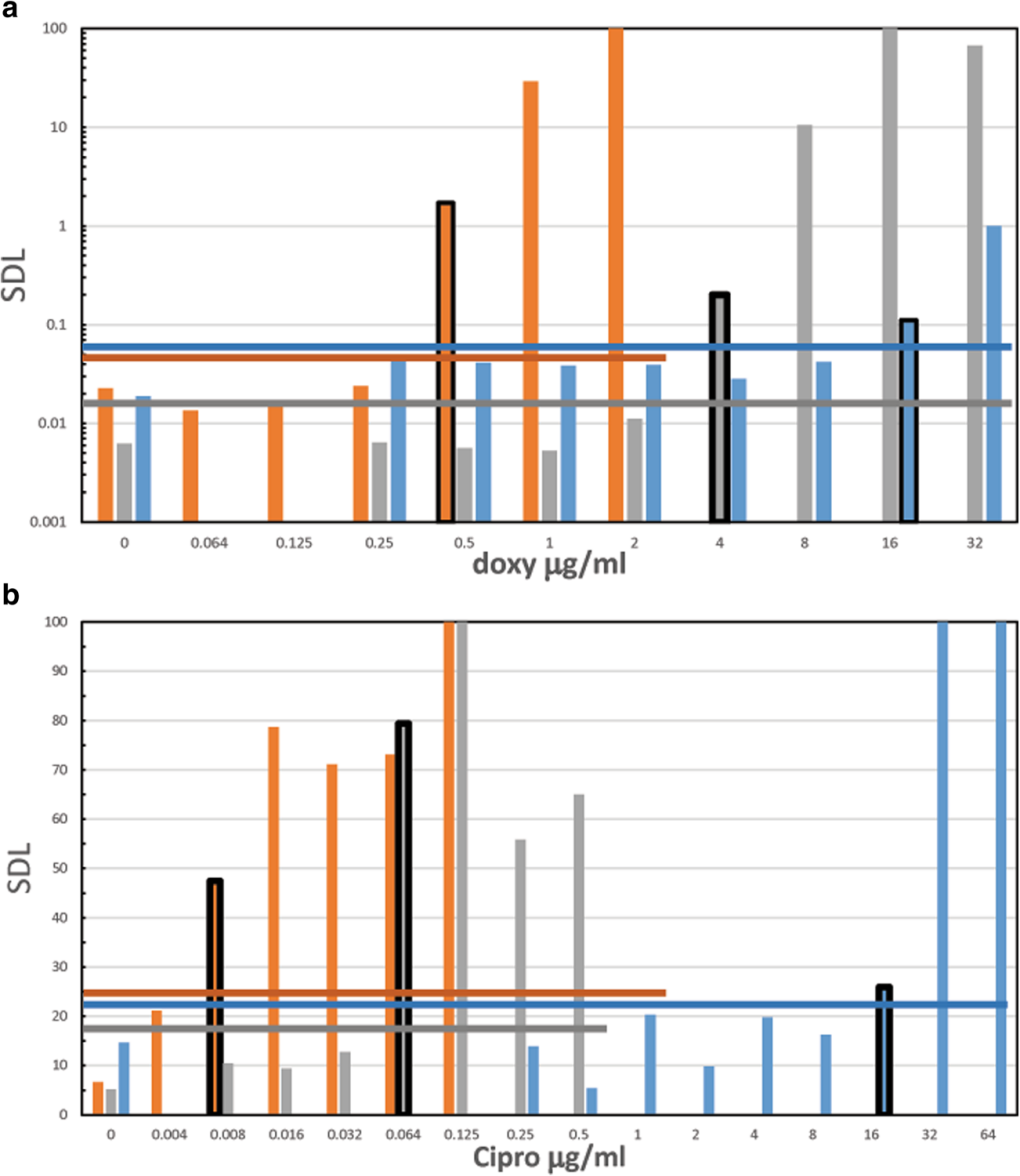
SDL overlay at 6 h measurements for different *Y. pestis* species with different antibiotic susceptibilities: **a** Doxycycline for the *Y. pestis* strains: EV76 (orange), Kim53Δ10Δ70–36 (gray) and Kim53Δ10Δ70–36-4 (blue) as sensitive, reduced sensitivity and resistant respectively. **b** Ciprofloxacin for the *Y. pestis* strains: Kim53Δ10Δ70 (orange), Kim53Δ10Δ70–42 (gray) and Kim53Δ10Δ70–66-6 (blue) as sensitive, reduced sensitive and not-sensitive respectively. For each SDL measurements there is its own threshold line for MIC determination. Threshold lines are in the same color as the correlated bars, MIC bar represented with black outline

Figure 7 shows the SDL values as a function of antibiotic concentration for Kim53Δ10Δ70 sub-strains with different susceptibilities to ciprofloxacin and doxycycline. This was done to test the applicability of the SDL method for discrimination between different bacterial susceptibility categories such as sensitive/non-sensitive/resistant. For the Doxycycline AST, we determined the MICs for 3 *Y. pestis* strains, the non-virulent sensitive strain EV76, the reduced sensitivity Kim53Δ10Δ70–36 and the resistant sub-strain Kim53Δ10Δ70–36-4. As can be seen on Fig. 7a we obtained the correct MICs (compared to standard method) of 0.5, 2 and 16 μg/ml respectively. For all three strains, the correct category was interpreted as sensitive, sensitive and resistant by CLSI category definitions. The same was done for Ciprofloxacin, as seen in Fig. 7b. We found for the non-virulent sensitive strain Kim53Δ10Δ70, its reduced sensitivity sub-strain Kim53Δ10Δ70–42 and for the Non Sensitive sub-strain Kim53Δ10Δ70–66-6 the correct MIC of 0.008, 0.125 and 16 μg/ml respectively. Again for all three, the correct category definition, sensitive, sensitive and non-sensitive is achieved according to the CLSI category, Table 3. Overall, we show that we can predict bacterial susceptibility to doxycycline and ciprofloxacin within 6 h of incubation.

## Summary and Conclusions

In this work, we present a rapid antibiotic susceptibility assessment method based on the change in spectral characteristics of the membrane fluorophore SynaptoGreen in live and dead bacteria using flow cytometry. This new concept of studying the spectral shift and intensity as a function of bacterial viability was analyzed using SIR and was further improved by the flow cytometry gate statistic to derive the values of SDL. This unique analysis enabled rapid determination of bacterial viability upon antibiotic exposure, including the determination of the bacterial MIC. This leads to a rapid AST with a time period relevant to clinical antibiotic treatment, namely, 4 to 6 h compared to the 24 to 48 h required in standard AST.

The evaluated MIC values obtained for the *Y. pestis* strains and antibiotics at all measured time increments are acceptable values compared to the standard microdilution reference method (Table 2). All MIC values meet the correct susceptibility category for the bacteria-antibiotic combinations (Table 3). It is important to note that after 24 h of exposure, as required by the standard microdilution test, all SDL-MIC values are in complete agreement with the MIC values obtained by the standard test. Our data show that we can obtain correct MIC values for the antibiotics measured here within 4 to 6 h. However, as shown with the reduced susceptibility strain, the category determination of non-sensitive vs. sensitive to ciprofloxacin can be determined after 6 h of incubation. This phenomenon probably represents the bactericidal activity that ciprofloxacin exerts on a bacterial population already at sub-MIC concentrations, which can be seen in the slower growth curves of cultures at sub-MIC concentrations [17]. Notably, at the correct reading time, all values were within one Log_2_ difference of the CLSI reference method [35], in compliance with FDA instructions for evaluating new susceptibility methods [38].

In summary, we have developed a unique analysis method to determine bacterial viability without the need for culture growth curve over time. Here, we utilized the technique for rapid prediction of the antibiotic susceptibility of the in vitro slow-growing bacteria *Y. pestis.* This new method is substantially different from many other fast detection methods since it does not require growth curve of the bacterial culture to assess viability. Viability is recorded directly from each bacterium by its unique interaction with the dye. This allows us to reduce AST incubation time to the minimal time required for antibiotic interaction with the bacteria, in contrast to the whole population behavior change monitored in ASTs, which are based on comparison to the growth control. By this method, we reduced the AST for *Y. pestis* from 24 to 48 h of incubation to only 4 to 8 h. This new rapid determination of antibiotic susceptibility is highly relevant clinically and could help reduce mortality and prevent the spread of the plague. Morever, this simple new method could probably be adopted also for other slow-growing clinically important bacteria.
